# Religion and sociodemographic characteristics at baseline of the Brazilian Longitudinal Study of Adult Health study

**DOI:** 10.1590/1806-9282.20230969

**Published:** 2024-03-15

**Authors:** Ana Carolina Varella, Itamar Souza Santos, Marcos Rafael Nogueira Cavalcante, Isabela Martins Benseñor, Paulo Andrade Lotufo

**Affiliations:** 1 Universidade de São Paulo, Center for Clinical and Epidemiological Research – São Paulo (SP), Brazil.; 2 Universidade de São Paulo, Department of Internal Medicine – São Paulo (SP), Brazil.

**Keywords:** Faith, Education, Religion, Epidemiology

## Abstract

**OBJECTIVE:**

The aim of this study was to investigate whether sex, age, race, income, education, and marital status are associated with having a religion in a sample of Brazilian men and women.

**METHODS:**

Data were obtained from 15,098 participants of the Brazilian Longitudinal Study of Adult Health, a longitudinal study that ultimately aims to investigate long-term outcomes of chronic diseases. The sociodemographic characteristics and data on religion status were self-reported during interviews conducted by trained personnel. All study procedures followed standard and validated protocols.

**RESULTS:**

There was a strong association between being a woman and having a religion (adjusted OR=2.12, 95%CI 1.95–2.31) when compared to men. Regarding age, those with 45–54 years were more likely to have a religion (adjusted OR=1.14, 95%CI 1.03–1.27). Blacks and Browns were more religious (adjusted OR=1.31, 95%CI 1.15–1.49, and OR=1.22, 95%CI 1.10–1.34, respectively) compared to Whites. Those with high income and education were less likely to state having a religion (adjusted OR=0.78, 95%CI 0.70–0.87, and adjusted OR=0.50, 95%CI 0.43–0.59, respectively). Those who did not have a stable conjugal union were found to be less religious (adjusted OR=0.82, 95%CI 0.75–0.89). Stratifying the analysis according to income showed that higher education was inversely associated with religion on both strata: lower and higher annual earnings.

**CONCLUSION:**

This study suggests that education is one of the most important socioeconomic characteristics to consider when studying religion. Race, sex, income, and marital status are also important factors; however, there was not a clear association between religion and age.

## INTRODUCTION

Religious involvement has been studied over the past decades, and researchers have attempted to identify the determinants of such an engagement^1^. Sociodemographic characteristics are considered as important determinants of religious beliefs^2^. Sex, age, race, income, education, and marital status have a crucial role on how one perceives their surroundings and builds their personal beliefs^3^. Evidence suggests that these factors might lead people to either more traditional or liberal meaning systems. People with more traditional meaning systems would tend to be more religious^4^.

According to the 2010 National Census, 92% of Brazilians stated having a religion (National Census, 2010)^5^. Even though the terminology "religion" does not reflect the religiosity or spirituality of an individual^6^, it still reflects some extent of the personal belief. In the past few decades, Brazil has suffered a religious transition, when people change their inherited beliefs and circulate among various religious affiliations, sometimes returning to the primary one^7^. This religious transition is probably associated with sociodemographic and economic changes as well^7^.

There are not many studies focusing on the relationship between sociodemographic characteristics and religion in the Brazilian population, and they often describe these characteristics by religious affiliation. Studying the characteristics of religious people helps elucidate how sociodemographic variables influence people's opinions and behavior toward religion^3^.

The Brazilian Longitudinal Study of Adult Health (ELSA-Brasil) is a cohort study including men and women from six different states in Brazil, with a socioeconomic gradient that grants good diversity to the sample allowing such investigation. The purpose of this study is to investigate whether sociodemographic characteristics are associated with religious status using baseline data.

## METHODS

### Study design and sample

Data were obtained from the baseline evaluation (August 2008 to December 2010) of ELSA-Brasil. It is a multicenter study located on six different cities of Brazil (Belo Horizonte, Porto Alegre, Rio de Janeiro, São Paulo, and Salvador e Vitória). At baseline, 15,105 civil servants between 35 and 74 years of age from institutions located in each of these cities were enrolled to participate. Exclusion criteria were as follows: intention to leave the institution at any time soon, severe cognitive or communication impairment, current or recent pregnancy (<4 months prior to the first interview), and if retired, reside outside of a study center's metropolitan area. ELSA-Brasil aims to study the incidence and associated risk factors of cardiovascular diseases and diabetes. The assessment consisted of interviews and clinical examinations carried out under strict quality control by trained personnel. The questionnaire covered a wide range of health-related topics, such as sociodemographic factors, cardiovascular risk factors, lifestyle, morbidity, cognitive function, medication use, mental health, and others^8,9^. More information about ELSA-Brasil can be found elsewhere^10-12^.

From the original 15,105 participants, we excluded missing values for the religion variable (7); therefore, the final overall sample was 15,098.

### Variables

Participants who responded yes to the question "Nowadays, do you have a religion?" were considered having a religion. Age was categorized as 35–44, 45–54, 55–64, and 65–74 years. Race was self-reported according to the Brazilian Census classification in the following categories: Black, Brown, White, Asian, and Indigenous. Marital status was dichotomized into without conjugal union (single, divorced, and widowed) or with conjugal union (married and others). Annual income was dichotomized using a cutoff value of US$ 20,000/year (at a rate of BRL 2.00=US$ 1.00), and education was categorized as less than high school, high school, and college.

### Statistical analyses

All statistical analyses were conducted with SPSS Statistics for Windows, version 24. The categorical variables were described using the chi-squared test and presented as absolute numbers and proportions. Logistic regression models were used to analyze the association between having a religion (dependent variable) and the sociodemographic variables of interest (sex, age, race, education, income, and marital status). Unadjusted and adjusted models were analyzed. All variables with a p<0.20 in Table 1 were included in the process. The final model included age, sex, education, income, race, and marital status as independent variables.

**Table 1:** Sample characteristics by religion status.

		Having a religion		
		No 3506 (23.2)	Yes 11592 (76.8)	p-value
Age, years				
	35–44	841 (24.0)	2497 (21.5)	0.001
	45–54	1287 (36.7)	4649 (40.1)	
	55–64	1006 (28.7)	3226 (27.8)	
	65–74	372 (10.6)	1220 (10.5)	
Sex				
	Men	2038 (58.1)	4846 (41.8)	<0.0001
	Women	1468 (41.9)	6746 (58.2)	
Race				
	White	2089 (60.8)	5697 (49.6)	<0.0001
	Brown	832 (24.2))	3370 (29.4)	
	Black	385 (11.2)	2012 (17.5)	
	Asian	100 (2.9)	273 (2.4)	
	Indigenous	32 (0.9)	125 (1.1)	
Education				
	Less than high school	300 (8.6)	1622 (14.0)	<0.0001
	High school	815 (23.2)	4418 (38.1)	
	College	2391 (68.2)	5552 (47.9)	
Income, US$/year				
	≤20,000	1002 (28.7)	5160 (44.7)	<0.0001
	>20,000	2489 (71.3)	6380 (55.3)	
Marital status				
	With conjugal union	2473 (70.6)	7999 (69.0)	0.081
	Without conjugal union	1032 (29.4)	3593 (31.0)	

As the results analyzing men and women separately were mostly similar, we do not present analyses stratified by sex. The significance level was set at 0.05.

## RESULTS

Table 1 shows that about 77% of participants stated having a religion. Among them, 58.2% were women, 40.1% were between 45 and 54 years of age, 17.5% were of Black race, 52.1% had up to high school of education, 44.7% had lower income, and 69% had conjugal union.

Results from the logistic regression models (Table 2) showed sex, education, income, race, and marital status to be independently associated with religion. Women were found to be more religious than men (adjusted OR=2.12, 95%CI 1.95–2.31). People in the age group of 45–54 years were found to be more religious compared to those with the age group of 35–44 years. There was no difference among the other age strata. Education was inversely associated with religion, especially for those who completed college or more (adjusted OR=0.50, 95%CI 0.43–0.59). Income was inversely associated with having a religion (adjusted OR=0.78, 95%CI 0.70–0.87) as well. Brown and Black people were found to be more religious when compared to White people (adjusted OR=1.22, 95%CI 1.10–1.34; OR=1.31, 95%CI 1.15–1.49, respectively). After adjustment for all variables, people without a conjugal union were found to be less religious (adjusted OR=0.82, 95%CI 0.75–0.89).

**Table 2:** Logistic regression models for the association between religion and sociodemographic variables.

		Unadjusted		Adjusted*	
		OR	95%CI	OR	95%CI
Sex					
	Men	Reference		Reference	
	Women^a^	1.93	1.79–2.09	2.12	1.95–2.31
Age, years					
	35–44	Reference		Reference	
	45–54	1.22	1.10–1.34	1.14	1.03–1.27
	55–64	1.08	0.97–1.20	1.08	0.96–1.21
	65–74^b^	1.11	0.96–1.27	1.23	1.06–1.43
Race					
	White	Reference		Reference	
	Brown^a^	1.49	1.36–1.63	1.22	1.10–1.34
	Black^a^	1.92	1.70–2.16	1.31	1.15–1.49
	Asian	1.00	0.79–1.27	0.91	0.72–1.16
	Indigenous	1.43	0.97–2.12	1.07	0.71–1.59
Income, US$/year					
	≤20,000	Reference		Reference	
	>20,000^a^	0.51	0.47–0.55	0.78	0.70–0.87
Education					
	Less than high school	Reference		Reference	
	High school and some college	1.00	0.87–1.16	0.97	0.83–1.13
	College or more^a^	0.43	0.38–0.49	0.50	0.43–0.59
Marital status					
	With conjugal union	Reference		Reference	
	Without conjugal union^a^	1.08	0.99–1.17	0.82	0.75–0.89

* Adjusted for age, sex, race, education, income, and marital status.

a p<0.0001.

b p<0.05.

Further analysis (supplementary Table 1) stratified by income and adjusted for sex showed that those with higher education were more likely to not have a religion (adjusted OR=0.33, 95%CI 0.24–0.46), while people of Black and Brown races were more likely to have it (adjusted OR=1.64, 95%CI 1.34–1.99, and adjusted OR=1.26, 95%CI 1.11–1.43, respectively), among those with higher income. For those with lower income, there were differences in religion status only for those with higher educational level (OR=0.76, 95%CI 0.61–0.94).

## DISCUSSION

This study showed an important inverse association between higher education and religion. A sensitivity analysis stratified by income showed higher education to be inversely associated with religion even for those with lower income. Women were more religious than men. Individuals of self-reported Black or Brown races were more frequently religious when compared to White individuals. Income was inversely associated with having a religion, and people with a conjugal union seemed to be more religious than those without one. Age was not clearly associated with religion.

We found women to be more religious than men, and this result remained significant after adjustment. Sex has been generally associated with religion, and women have been reported to be more religious than men^13,14^. Data from a survey conducted by the Committee on the Social and Psychological Factors Affecting Fertility in Indianapolis (US) reported greater interest in religion and religious practices among women when compared to men^15^. Our findings extend these results to a more diverse sample in terms of race. Additionally, in a cross-sectional study using data from the Sexual Behavior of the Brazilian Population and HIV/AIDS Perceptions Study in Brazil, Almeida & Monteiro^7^ showed that a higher proportion of men considered themselves as not having a religion compared to women.

Age has been pointed out as important for religious engagement^16^. Analyzing the report by Almeida & Monteiro^7^, we see that people under 25 and over 41 years of age were the most religious groups, showing an unclear yet discussible pattern of association. Younger people might still carry their parents’ beliefs, followed by a life period when individuals experience other religious expressions. Possibly, in older ages, individuals decide to return to the primary belief or stay with the new one. A different unclear pattern was found in our study. We found that people between 45 and 54 years of age were more religious when compared to the youngest. Surprisingly, older age strata were not associated with having a religion in this sample. Religious dynamics in young adults cannot be fully explored with ELSA-Brasil data, as participants in our cohort aged at least 35 years at baseline. However, this study's results reinforce the idea that age is not a reliable predictor for religious status^1^. Age and religion relationship might be associated with religious affiliation and family beliefs^17^.

This study also showed that having a conjugal union was positively associated with religious status after multivariate adjustment. The association between marital status and religion has been controversial. A study by Mormons reported no effect of marital status on religious behaviors^2^. However, Fisher^18^ showed that marital status influences the nature of people's social relationships, and Cornwall^2^ also showed that it could influence the social relations within a religious group. These latter findings could help to explain the nature of our results. It is suggested that marital status itself would not lead people to be more religious but could influence how they find groups to engage, such as more traditional environments that tend to value religious principles^4^.

Another sociodemographic characteristic known to play an important role in religious beliefs is race^19,20^. We found Black and Brown people to be more religious than White people, and among all the three, people of Black race were the most religious group. Studies suggest a historical explanation for these findings, Black people would lean toward faith and get involved in religious groups in order to have comfort and help from a local community, once they historically have weaker political and societal support when compared to White people^17,21^. We found no difference in Asian and Indigenous people, which could be explained by the smaller sample size of those two races.

Our results showed a strong inverse association between education and having a religion, that is, the higher the educational level (college or more), the lower the frequency of people identifying themselves with a religion. Brazil is primarily a Christian country (Catholics and Protestants)^7^, and these religious denominations are usually associated with lower educational levels. This present study showed that higher income was also associated with a lower frequency of people stating to have a religion. Some studies suggest that people in higher educational and socioeconomic levels tend to incorporate beliefs that shift toward the intellectual meaning instead of relying on faith^1,4^.

Higher education usually means higher income, which is concentrated primarily among people of White race in Brazil^22^. Attempting to capture the possible relationship among those three characteristics and their influence on religion, we conducted a sensitivity analysis among participants with lower and higher incomes. Among those with higher income, the Black and Brown races were positively associated with religion, while higher educational level was inversely associated with having a religion. For those with lower income, the differences in religion status were seen only for those with higher educational level, and this could mean a strong association between religion and education independent of income.

Some limitations of this study include the fact that all variables were self-reported which could introduce some bias. Also, ELSA-Brasil was not meant to investigate religious involvement. Therefore, the variables included in the baseline assessment did not reflect the spirituality of the individual, but they still reproduce some extent of the personal belief, as some authors mentioned that there is still an overlap between the terms "religion" and "spirituality" for many people^23^.

## CONCLUSION

Among all sociodemographic factors studied, education seemed to have greater influence on religious status, followed by race, income, sex, and marital status. A clear trend of association with age was not observed.

## Supplementary Material


