# Supplementary material for: Religion and sociodemographic characteristics at baseline of the Brazilian Longitudinal Study of Adult Health study
Source: Rev Assoc Med Bras (1992). 2024 Mar 15;70(1):e20230969. doi: 10.1590/1806-9282.20230969 (PMC10941871; doi:10.1590/1806-9282.20230969)
Supplement: Supplementary file 1 [file 1806-9282-ramb-70-1-e20230969-Suppl01.docx]

**Supplementary Table 1**. Logistic regression models for the relationship between religion and education, and race, stratified by income.

|  | **Unadjusted** | | **Adjusted*** | |
| --- | --- | --- | --- | --- |
|  | **OR** | **95%CI** | **OR** | **95%CI** |
| **Lower income** |  |  |  |  |
| **Race** |  |  |  |  |
| White | Reference |  | Reference |  |
| Brown | 0.98 | 0.84–1.16 | 1.01 | 0.86–1.19 |
| Black | 1.05 | 0.88–1.25 | 1.00 | 0.84–1.21 |
| Asian | 1.21 | 0.68–2.15 | 1.17 | 0.65–2.09 |
| Indigenous | 0.88 | 0.52–1.48 | 0.91 | 0.54–1.55 |
| **Education** |  |  |  |  |
| Less than high school | Reference |  | Reference |  |
| High school and some college | 0.97 | 0.82–1.14 | 0.89 | 0.75–1.05 |
| College or more | 0.90 | 0.73–1.11 | **0.76** | **0.61–0.94** |
| **Higher income** |  |  |  |  |
| **Race** |  |  |  |  |
| White | Reference |  | Reference |  |
| Brown | 1.27 | 1.12-1.43 | **1.26** | **1.11–1.43** |
| Black | 1.74 | 1.43-2.12 | **1.64** | **1.34–1.99** |
| Asian | 0.95 | 0.73-1.24 | 0.85 | 0.65-1.11 |
| Indigenous | 1.00 | 0.54-1.87 | 1.09 | 0.60-2.06 |
| **Education** |  |  |  |  |
| Less than high school | Reference |  | Reference |  |
| High school and some college | 1.00 | 0.71-1.42 | 0.86 | 0.60-1.22 |
| College or more | **0.39** | **0.28–0.55** | **0.33** | **0.24–0.46** |

*Adjusted for sex.
